# Primary ALK-positive anaplastic large cell lymphoma of the urinary bladder in a pediatric patient: a case report and review of literature

**DOI:** 10.1007/s12308-026-00737-y

**Published:** 2026-09-26

**Authors:** Navdeep Kaur, Akhila Aravind, Sarika P. Jain

**Affiliations:** 1 Department of Pathology, University of New Mexico, Albuquerque, NM 87106 USA; 2 Department of Pathology, University of Mississippi Medical Center, Jackson, MS 39216 USA

**Keywords:** Bladder, Pediatric, ALK-positive anaplastic large cell lymphoma, CD30

## Abstract

**Background:**

Primary lymphomas of the urinary bladder are rare, most of which are low-grade B-cell lymphomas. Primary anaplastic large cell lymphoma (ALCL) with bladder involvement is extremely rare, with most cases seen in adult patients. Here, we report a case of ALK-positive ALCL in a 14-year-old male.

**Case presentation:**

A 14-year-old male presented with several months of gross hematuria that had become painful. Computed tomography (CT) urogram showed a mass-like thickening of the anterior bladder wall. Cystoscopy showed a 3.5-cm pedunculated lesion at the bladder dome; transurethral excision was performed. Histology revealed sheets of large pleomorphic cells with horseshoe-shaped nuclei and brisk mitotic activity. Tumor cells were positive for CD4, CD5, and CD30 (diffuse/strong) and negative for CD45. ALK immunostain showed nuclear and cytoplasmic staining. A diagnosis of ALK + ALCL was made. Staging CT was negative for disease. The bone marrow was uninvolved. The patient received multi-agent chemotherapy including brentuximab vedotin. A 2-month follow-up cystoscopy showed a well-healed resection site. Seven months after completing therapy, the patient had recurrent hematuria. Cystoscopy revealed an ulcerated lesion, but biopsies were reactive and showed no lymphoma. The patient remains under surveillance with urology and oncology.

**Conclusion:**

Primary bladder ALK + ALCL is exceedingly rare in children. Uniform CD30 positivity and ALK positivity are the key diagnostic clues.

## Introduction

Anaplastic large cell lymphoma (ALCL) is a rare subtype of peripheral T-cell lymphoma characterized by strong and uniform CD30 expression in the neoplastic cells [1]. Based on anaplastic lymphoma kinase (ALK) expression, systemic ALCL is classified into ALK-positive and ALK-negative subtypes, which differ in age distribution, molecular features, and clinical behavior [2]. ALK-positive ALCL is more commonly seen in children, adolescents, and young adults, whereas ALK-negative ALCL typically affects older patients [3].

ALCL most often presents with lymph node involvement. Extranodal disease is also common; frequently involved extranodal sites include the skin, bone, soft tissue, lung, liver, and bone marrow [5]. Involvement of the urinary bladder, however, is extremely rare. Primary lymphoma of the bladder itself is uncommon, accounting for approximately 0.2% of extranodal lymphomas, and the majority of reported cases represent extranodal marginal zone lymphoma or diffuse large B-cell lymphoma [6, 7]. ALCL involving the bladder has been reported only rarely, mostly as isolated case reports or small case series [8–10].

In the pediatric population, ALK-positive ALCL constitutes a significant proportion of T-cell lymphomas and accounts for approximately 10–15% of non-Hodgkin lymphomas [3, 4]. It shows a male predominance and frequently presents with advanced-stage disease. ALK positivity is associated with a relatively favorable prognosis when compared with other peripheral T-cell lymphomas, with a good response to combination chemotherapy and reported long-term survival rates of 70–90% [4, 5].

The clinical presentation of bladder ALCL is nonspecific and overlaps with more common urologic conditions. Patients typically present with hematuria, dysuria, urinary frequency, or pelvic pain [6, 8]. As bladder tumors are most commonly of epithelial origin, lymphoma is often not considered initially. On histology, ALCL may closely mimic poorly differentiated urothelial carcinoma, sarcoma, inflammatory myofibroblastic tumor, metastatic carcinoma, or melanoma, particularly on limited biopsy material. Therefore, diagnosis is challenging and requires careful correlation of morphology with immunohistochemistry and molecular studies, including demonstration of CD30 positivity, ALK expression, and clonal T-cell receptor gene rearrangement [5, 8, 9].

Review of the available literature indicates that ALK-positive ALCL involving the urinary bladder has been reported predominantly in adults, most often young to middle-aged males, and in association with systemic disease [8–10]. Primary bladder ALCL without systemic disease is extremely rare. To date, only two cases of ALK-positive ALCL primarily involving the urinary bladder have been reported [9]. Pediatric cases are exceptionally rare.

In this report, we describe a second case of ALK-positive anaplastic large cell lymphoma involving the urinary bladder in a pediatric patient. This case further expands the clinicopathologic spectrum of this rare entity and highlights the importance of considering lymphoma in the differential diagnosis of unusual bladder masses in children, as well as the need for a comprehensive pathologic workup to establish an accurate diagnosis.

## Clinical history

A 14-year-old male presented with a several-month history of gross hematuria which recently worsened and became painful. He had no prior history of lymphoma or tumors. He denied fevers, night sweats, weight loss, or appetite changes.

### Imaging and endoscopy

CT urogram showed minimal perivesical stranding, focal thickening of the left anterolateral bladder wall with partial intraluminal extension. The overall appearance was concerning for an underlying urinary bladder neoplasm. Hence, further evaluation with cystoscopy was advised. The cystoscopic examination revealed a 3.5-cm pedunculated mass arising from the dome of the urinary bladder. Complete transurethral excision of the bladder wall mass was subsequently performed.

### Pathology

A 5.5 cm aggregate of tan-pink tissue fragments was received for histopathologic evaluation. Microscopic analysis of formalin-fixed paraffin-embedded tissue showed sheets of large, atypical cells with irregular nuclear contours, vesicular chromatin, prominent nucleoli, and frequent mitoses. Many “hallmark” cells with horseshoe-shaped nuclei were seen.

**Fig. 1:**
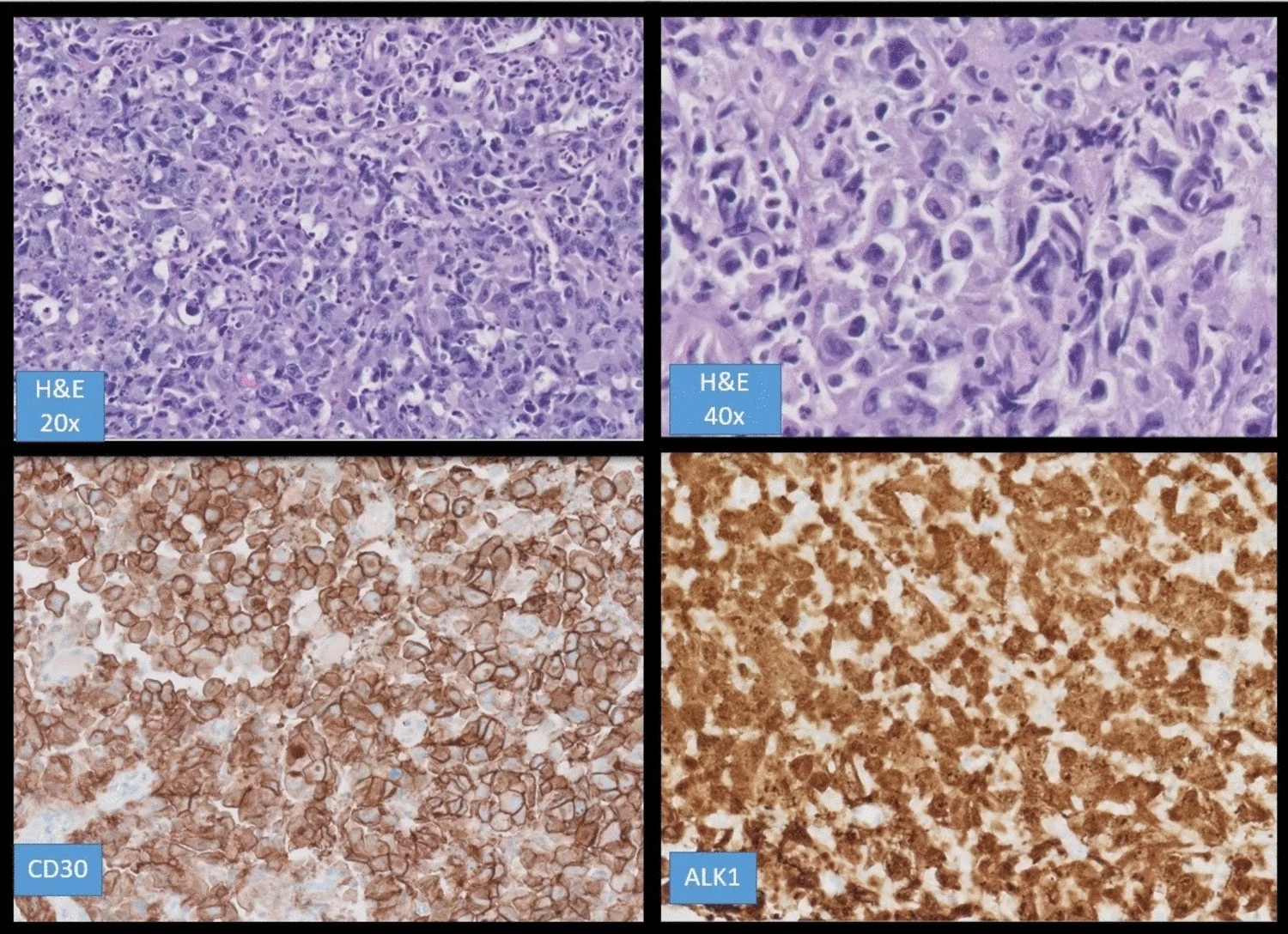
H&E sections show large atypical cells with corresponding immunohistochemical staining demonstrating positivity for CD30 and ALK1

Immunohistochemical stains (IHC) demonstrated diffuse/strong positivity for CD30 in the large cells. ALK1 showed nuclear and cytoplasmic positivity (Fig. 1). CD43 showed patchy positivity in tumor cells. The neoplastic cells were also positive for CD4 and CD5, while CD45, CD3, CD7, and CD8 were negative. CD20 highlighted a few background B-lymphocytes. Additional immunostains including CD31, CD34, CD68, desmin, smooth muscle actin (SMA), p63, S100, and pancytokeratin were negative.

### Staging

Contrast-enhanced CT of chest, abdomen, and pelvis showed no nodal or visceral disease. Bone marrow biopsy was normocellular without lymphoma. PET-CT showed no FDG avid extra pelvic disease.

### Treatment and follow-up

The patient subsequently received systemic chemotherapy approximately 1 month following surgical resection. His treatment was using an ALCL99-based backbone integrated with brentuximab vedotin (an antibody–drug conjugate that targets the CD30 protein universally expressed on ALCL cells) per COG ANHL12P1 protocol with additional agents including ifosfamide, high-dose methotrexate, cytarabine, etoposide, and dexamethasone. He tolerated therapy well. Two months post-initiation, cystoscopy showed a well-healed site with no residual or recurrent tumor. At 7 months off therapy, hematuria recurred; cystoscopy showed an ulcerated lesion, but biopsies revealed reactive changes only.

The patient has continued to undergo surveillance with urology and oncology and, at the most recent follow-up approximately 3 years after diagnosis, remains clinically well and disease-free with no evidence of recurrent ALCL.

## Discussion

Primary lymphoma of the urinary bladder is rare, accounting for approximately 0.2% of extranodal lymphomas, and most cases are of B-cell origin, most commonly extranodal marginal zone lymphoma of mucosa-associated lymphoid tissue or diffuse large B-cell lymphoma [6, 7]. T-cell lymphomas involving the bladder are rare, and ALK-positive anaplastic large cell lymphoma (ALK + ALCL) at this site has been reported only sporadically in the literature [8–10]. Most published cases involve adults, making bladder involvement by ALK + ALCL in the pediatric population exceedingly uncommon.

ALK-positive ALCL is a distinct clinicopathologic entity characterized by strong CD30 expression and ALK gene rearrangement; it most often shows NPM1:ALK rearrangement, resulting in nuclear and cytoplasmic ALK staining on immunohistochemistry [1, 5]. It predominantly affects children, adolescents, and young adults and constitutes a significant proportion of pediatric T-cell lymphomas [3, 4]. While nodal disease is typical, extranodal involvement is common, particularly involving the skin, bone, soft tissue, lung, liver, and bone marrow [5]. Involvement of the urinary bladder, however, remains extremely rare, likely contributing to delays or difficulty in diagnosis.

Bladder ALCL presents with nonspecific urinary symptoms such as hematuria, dysuria, urinary frequency, or pelvic pain, which overlap with other more common urothelial neoplasms and inflammatory conditions [6, 8]. As a result, lymphoma is often not initially considered in the differential diagnosis, particularly in pediatric patients where bladder tumors themselves are rare. In our case, the patient presented with gross hematuria and a bladder mass, raising initial concern for a primary urothelial or mesenchymal neoplasm.

Histologically, ALCL poses a diagnostic challenge due to its pleomorphic morphology and ability to mimic poorly differentiated urothelial carcinoma, sarcomatoid carcinoma, embryonal rhabdomyosarcoma, inflammatory myofibroblastic tumor, metastatic melanoma, or other high-grade malignancies [8, 9]. The presence of classic “hallmark” cells with horseshoe-shaped or reniform nuclei is helpful but may not be prominent in small or fragmented biopsy specimens. Therefore, a broad immunohistochemical panel is essential.

Uniform strong CD30 expression together with ALK positivity remains the most important diagnostic clue for ALK + ALCL [1, 5]. CD45 is usually positive in ALCL but may be weak or negative, which can further complicate distinction from carcinoma or classical Hodgkin lymphoma. In such cases, correlation with morphology, ALK staining pattern, and supportive markers such as EMA and cytotoxic markers (TIA, granzyme B, perforin) and lineage/TCR studies is critical [5, 8, 9]. ALK immunostaining also provides indirect information regarding the underlying fusion partner. Nuclear and cytoplasmic staining, as seen in our case, is characteristic of NPM1:ALK rearrangement, whereas purely cytoplasmic or granular staining patterns suggest alternative fusion partners [5].

Review of the literature indicates that most reported cases of bladder ALK + ALCL have occurred in adults, often young to middle-aged males, and frequently in the setting of systemic disease [8–10]. Pediatric cases are exceptionally rare. To date, only one pediatric case of ALK-positive ALCL involving the bladder has been documented, making the present case the second reported pediatric example to our knowledge [9]. This underscores both the rarity of this presentation and the importance of awareness among pathologists and clinicians.

Accurate staging is essential once the diagnosis is established. In our case, comprehensive staging showed no evidence of nodal or visceral involvement, supporting primary bladder disease. ALK-positive ALCL is generally chemosensitive and associated with a favorable prognosis compared with other peripheral T-cell lymphomas [4, 5]. Our patient received treatment as per ANHL12P1-Arm BV. Although our patient has remained disease-free for approximately 3 years, this case provides additional evidence that durable remission can be achieved in pediatric ALK-positive ALCL involving this rare extranodal site with contemporary multimodal therapy.

The clinical course in our case also highlights the importance of careful post-treatment surveillance. Recurrent hematuria after completion of therapy raised concern for relapse; however, biopsy showed only reactive changes. Post-treatment inflammatory or ulcerative bladder changes can closely mimic recurrence both clinically and endoscopically, emphasizing the need for histologic confirmation before initiating further therapy.

In summary, ALK-positive ALCL involving the urinary bladder is an exceptionally rare entity in children. Awareness of this diagnosis, recognition of its characteristic immunophenotype, and correlation with clinical and imaging findings are essential to avoid misdiagnosis. Early and accurate diagnosis allows timely initiation of appropriate systemic therapy, which is associated with excellent outcomes in most patients.

## Conclusion

Pediatric primary bladder ALK + ALCL is extraordinarily rare. When a child presents with hematuria and a bladder mass, a lymphoma (including T-cell/ALK-rearranged) should be in the differential. Strong CD30 with ALK positivity, particularly a nuclear + cytoplasmic ALK pattern, supports ALK + ALCL, although CD45 may be reduced/negative.

## Data Availability

No datasets were generated or analysed during the current study.
